# Study Protocol: Gut microbiota profiles implicated in the onset of autism spectrum disorders in preterm infants: A two-year follow-up study

**DOI:** 10.1192/j.eurpsy.2022.706

**Published:** 2022-09-01

**Authors:** P. Navalón, F. Ghosn, B. Almansa, I. Lara, A. Pinilla, Á. Solaz, C. Zapata De Miguel, Y. Cañada, A. García-Blanco

**Affiliations:** 1 La Fe Health Research Insitute, Neonatal Research Group, Valencia, Spain; 2 La Fe Health Research Insitute, Mental Health Research Group, Valencia, Spain

**Keywords:** Autism Spectrum Disorders, microbiota, prematurity, follow-up study

## Abstract

**Introduction:**

Preterm infants are at high-risk of developing autism spectrum disorders (ASD). The underlying mechanisms that explain the link between prematurity and ASD are unclear. Perinatal environmental factors may disrupt the gut-brain communication, when the gut microbiome composition is established and brain programming occurs. Therefore, the disruption of the gut-brain axis communication in response to perinatal environmental events may shed light on the association between prematurity and ASD.

**Objectives:**

To describe a new research project protocol which aim is to develop a dynamic model of gut microbiota variation in response to environmental factors that modulate the ASD risk in preterm infants.

**Methods:**

A two-year prospective observational study will be carried out, in which preterm infants will be assessed at birth, 40th postmenstrual week, at 6, 12, and 24 months of corrected age. Two-hundred preterm infants will be recruited. A comprehensive assessment will be conducted by collecting data on sociodemographic characteristics, medical history, family functioning, neurodevelopment, ASD screening, and diagnosis. Microbiome composition and microbial activity will be determined from feces.

**Results:**

The expected results are: i) to characterize ASD since its early manifestations in an at-risk population, allowing an early diagnosis and intervention to improve clinical outcomes; ii) to identify early microbiota biomarkers in order to find potential pathophysiological pathways; iii) to understand the protective and risk factors associated to ASD since perinatal period.

**Conclusions:**

A two-year predictive model will be generated based on environmental and gut microbiota variables. This predictive model of ASD would allow prevention, early diagnosis, improvement of prognosis, and personalized treatments in preterm infants.

**Disclosure:**

No significant relationships.

